# Basal Cell Carcinoma: From the Molecular Understanding of the Pathogenesis to Targeted Therapy of Progressive Disease

**DOI:** 10.1155/2011/650258

**Published:** 2010-12-29

**Authors:** Daniela Göppner, Martin Leverkus

**Affiliations:** ^1^Department of Dermatology and Venerology, Otto-von-Guericke-University Magdeburg, Leipziger St. 44, 39120 Magdeburg, Germany; ^2^Department of Dermatology, Venerology, and Allergology, Medical Faculty Mannheim, Ruprecht-Karls-University of Heidelberg, Theodor-Kutzer-Ufer 1-3, 68167 Mannheim, Germany

## Abstract

Due to intensified research over the past decade, the Hedgehog (HH) pathway has been identified as a pivotal defect implicated in roughly 25% of all cancers. As one of the most frequent cancer worldwide, the development of Basal cell carcinoma (BCC) due to activation of the HH pathway has been convincingly demonstrated. Thus the discovery of this central tumor-promoting signalling pathway has not only revolutionized the understanding of BCC carcinogenesis but has also enabled the development of a completely novel therapeutic approach. Targeting just a few of several potential mutations, HH inhibitors such as GDC-0449 achieved already the first promising results in metastatic or locally advanced BCC. This paper summarizes the current understanding of BCC carcinogenesis and describes the current “mechanism-based” therapeutic strategies.

## 1. Introduction

BCC, the most commonly diagnosed skin cancer in persons of fair complexion, has become the focus of intensified translational debate lately. Following the circumstantial evidence that ewes gave birth to cyclopic and malformed lambs after nibbling on Veratrum californicum, a Corn lily, the causative teratogenic compound, cyclopamine, was discovered [1, 2]. Increased research on this agenda and the understanding of its functioning led to the discovery of the Hedgehog signalling pathway (HH) as an essential cascade in embryonic development [3]. Proof of a specific mutation in BCC's Hedgehog pathway showed for the first time that an aberrant HH signalling is also strongly implicated in cancerogenesis of skin tumors [4]. Though a wide range of efficient therapeutic options are well established in the treatment of sporadic BCC, the newly developed HH inhibitors and first study results give rise to a curative or even secondary-prophylactic approach in hereditary, advanced, or even metastatic variants. 

This paper summarizes the current knowledge of clinical aspects and the molecular pathogenesis of this form of skin cancer. Moreover, we discuss current and future therapies that are needed in order to allow efficient treatment of BCC in complicated localization, in patients with multiple tumors or genetic disease predisposing for BCC development, or patients that are not eligible for surgery.

## 2. Epidemiology and Clinical Aspects

**Figure 1:**
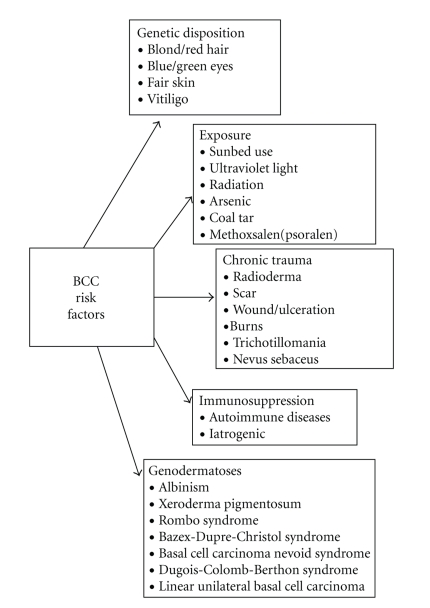
Risk factors of BCC, adapted from Rubin et al. [16].

First described by Krompecher in 1900 as “carcinoma epitheliale adenoides” [5] and named after its morphological affinity to the normal cell of the basal layer, BCC is the most common keratinocyte skin cancer (KSC) in persons of Caucasian ancestry. Although it presumably develops from epidermal stem cells of the outer root sheat of the hair follikel, the precise origin of BCC is still unknown thus far [6, 7]. Its incidence is estimated up to 100 cases per 100,000 and even higher depending on geographical or complexion disparities. Hence, BCC as well as other KSCs are often excluded from cancer-registry statistics, thereby underestimating the socioeconomic burden of this form of cancer [8–10]. More common in men than in women, BCC usually arises at an average age of 60 years. Apart from the environmental exposure to arsenic, ionizing radiation, oral methoxsalen (psoralen), and immunosuppressive therapy such as in organ transplant recipients [11, 12], persons with a fair skin type-I complexion (including red or blonde hair, light coloured eyes, freckling) and people with a history of intermittent sun exposure and severe sunburn during childhood are at highest risk [13]. In particular ultraviolet (UV) irradiation in inverse correlation with reduced or impaired skin pigmentation is generally considered to be the major risk factor of basal cell carcinoma [14, 15]. Depending on timing (childhood, adolescence), pattern (intermittent, continuous), source (natural, artificial), and amount (cumulative sun exposure), its impact on BCC development is, however, far more complex and needs further detailed study [16]. Though the rates are still highest for the naturally sun exposed skin of elderly man, the trend over the past decade is clearly towards an increasing incidence of BCC in younger women due to excessive tanning and sunbed use (Figure 1) [17].

The majority of sporadically occurring BCCs arise in sun-exposed areas with over 80% of all cases developing on the head and neck. Unlike squamous cell carcinoma (SCC), BCCs do not have detectable precursor lesions and usually present themselves *de novo* as a palpable, localised, translucent tumour with overlying teleangiectasias. For hitherto unknown reasons, they differ in three main clinical as well as histological phenotypes: the nodular BCC exhibiting a pearly rolled border at times with central crusting and ulceration, the superficial subtype with its scaly erythematous patch or plaque-like appearance and the sclerosing, infiltrative, or morpheaform variant that clinically presents as a scar-like, centrally atrophic, whitish, indurate tumour with indistinct margins. Frequently, those three histological subtypes are mixed. In addition to aggressive BCCs such as the infiltrative, micronodular, or basosquamous subtypes, uncommon BCC variants include the clear-cell, granular-cell, or adamantinoid variants, and adnexal differentiation. Pigmented tumours, known to carry p53 mutations [18], may mimic several differential diagnoses including melanoma and therefore need to be confirmed by biopsy. Although erosion and ulceration can develop quite early, especially in the nodular variant, sporadic BCC is in general a slow growing, delayed infiltrating, or destructive tumour that, even in view of other risk factors in terms of a large diameter >2 cm, incomplete incision and perivascular involvement, metastases only occur after years of existence in 0.55% of all cases [19]. Once metastasised in regional lymph nodes followed by bone, liver, and lung, the prognosis is poor with a mean survival of at most 3.6 years after diagnosis [19, 20]. 

In contrast to the sporadic variant of BCC, a hereditary disorder, also known as Gorlin syndrome or basal cell nevus syndrome (BCNS), exhibits a marked propensity to develop numerous BCCs already during adolescence and occasionally even in childhood. As an autosomal dominant inherited genodermatosis with an estimated incidence of 1 : 150 000 in the general population, BCNS is very rare. It is characterized by a range of developmental anomalies—most notably in the head and neck area that allowed the oral pathologist and dentist Robert Gorlin to describe it first - and a predisposition to various other forms of cancers. Apart from skeletal abnormalities such as splayed ribs, Sprengel and pectus deformity, these patients suffer from ectopic calcification, odontogenic keratocysts, facial dismorphism with macrocephaly, palmoplantar pits and tumours in terms of cardiac and ovarian fibroma, meningeoma, medulloblastoma, rhabdomyosarcoma, mesenteric cysts, and other neuroectodermal tumours [21]. Most prominent among these clinical findings is the early and very strong disposition to develop several, occasionally hundreds of BCCs, especially after radiation given for treatment of progressive BCC or medulloblastoma. It was, however, the intensified research on BCNS with proof of its cause, a mutated PTCH1 gene in the majority of cases, that linked cancer to the HH signalling pathway for the first time in 1996 [4, 22].

## 3. Molecular Pathogenesis

### 3.1. Hedgehog Signalling Pathway

**Figure 2:**
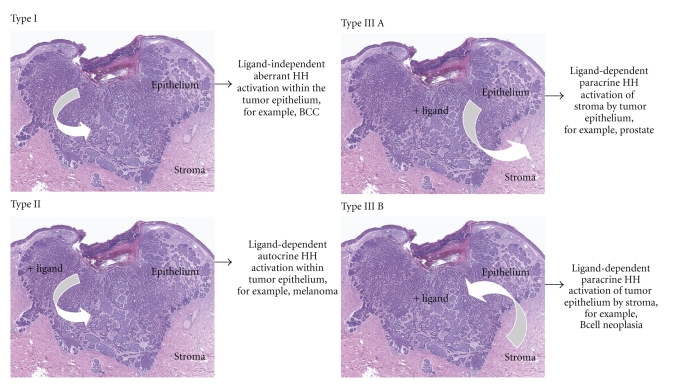
HH pathway model systems in cancer, adapted from Scales and de Sauvage [26].

The hedgehog (HH) family of intercellular signalling proteins play a pivotal role in many fundamental processes of embryogenic development. They are central to differentiation, growth, pattering, morphogenesis, and function of different cells and organs as well as epithelial and mesenchymal tissue interactions in vertebrates and invertebrates alike [23, 24]. Malfunction or mutation of these proteins lead to substantial impairment as already shown by the prickly, hedgehog-like appearing of mutant flies of Drosophila melanogaster after which the family of proteins was named. Probably by duplication of a single-ancestral gene, mammalians, in contrast to invertebrates with just one HH gene, develop three different types of homologs: the Sonic, the Desert, and the Indian type. The HH pathway is initiated whenever one of these ligands binds and thereby inactivates the transmembrane tumour-suppressor protein patched homologue 1 (PTCH-1). As a consequence, PTCH-1 then permits its receptor smoothened (SMO), another transmembrane protein, to transmit signals to downstream targets by means of the GLI family of transcription factors. Under normal conditions, and mostly in adults, the hedgehog pathway is ligand dependent and actively repressed because PTCH-1 constantly inhibits SMO, the key activator of the GLI pathway. Especially Sonic hedgehog (SHH), as the most widely characterized signalling pathway of the three types, provides a unique example of how the same molecular cascade leads to different pattering in different tissue types solely by distinct transcriptional programs based upon its local concentration [25]. Inappropriate activation due to mutations within this cascade however was clearly identified by a growing body of evidence to be a pivotal cause of carcinogenesis, in particular, in BCNS-associated BCCs and medulloblastoma. According to Scales de Sauvage, so far three different model systems are proposed on how the HH pathway is involved in the generation of different types of cancer (Figure 2) [26].

### 3.2. Mutations of Hedgehog Signalling Pathway in BCC

Independent of the underlying oncogenic mutation, in nearly all sporadic as well as BCNS-linked BCCs, uncontrolled stimulations of the hedgehog signalling are found [27, 28]. Due to relatively stable genomes when compared to other extracutaneous cancer, BCCs routinely carry mutations in 30% to 50% of the tumors in p53 or PTCH-1 [29–31]. The latter either looses thereby its function (loss of function mutation) or less commonly activates SMO (gain of function mutation) [4, 22, 32]. Continuously stimulated by SMO, a variety of cell-specific target genes then interfere with the physiological function via endothelial growth factor and angiopoetin (resulting in angiogenesis, cell proliferation, metastasis, and cell survival), ultimately leading to cancer [26, 33]. SMO itself is mutated only in 10% of all sporadic BCCs [29]. A few other alternations of the HH pathway, for example, in SHH or GLI, have been tried to be identified but could not be confirmed so far [29, 34].

### 3.3. Genetic Predispositions, Mutations and Interacting Pathways

In view of the known complex interplay of genes and epigenetic and environmental influences in carcinogenesis, the development of BCC and cancer in general is, however, certainly far more complex and cannot possibly be reduced to three somatic mutations within the hedgehog pathway. With the focus on the downstream target genes and effects of HH signalling, the BCC carcinogenesis probably constitutes an intricate mechanism of several interacting pathways and mutated genes that regulate pigmentation, DNA repair, and apoptosis. Many other mutations have been proven to be implicated in BCC so far. Especially the sequence of downstream mediators in HH seems to differ in various tissues. Several, such as CD95, BCL-2, PDGFR*α*, or cFLIP, are currently under investigation [35]. Furthermore, contributions of the FOX gene family, in particular FOXM1 and FOXE1, appear to be involved in downstream signalling [35–38]. As HH target genes, both FOX proteins control for a normal mitosis and are overexpressed in BCC in comparison to normal keratinocytes [39, 40]. But it is not yet understood which changes are crucial in BCC and therefore represent “drivers” but not “passengers” during tumorigenesis of BCC [14, 41]. 

A similar lack of knowledge still exists for the interaction of the GLI signalling pathway with other cellular signals. The Phosphoinositol-3-kinase (PI3K) cascade interacts with SHH in at least two ways. While it inhibits protein kinase A (PKA-) mediated phosphorylation, it also stabilizes GLI2. On the other hand, SHH activates PI3K, for example, in prostate cancer [42]. But up to now, no proof for PI3K involvement in BCC carcinogenesis could be given [14]. The relationship of the Ras/Raf signalling pathway and BCC is less well defined [29]. In comparison, the obvious requirement of Wnt signalling in the downstream activation of HH for these tumours [43] hints at novel possibilities to the therapeutic approach in BCC in addition to HH inhibitors (described below) [14]. 

From a clinical point of view, the most convincing yet rather confusing—due to several contradicting results—research focuses on the association of BCC with pigmentation and DNA repair genes, respectively. At least for sporadic BCCs as the classic UV-induced variant and those that arise in patients with xeroderma pigmentosum (XP), a frequent type of PTCH1 and p53 mutations could be identified [44]. 

The clinical presumption that the increased incidence of BCC in elderly could be a consequence of diminished DNA repair due to aging seems therefore not so farfetched [45]. Hence, the repair of UV-induced damage should reduce BCC development [44, 46], although the use of sunscreens failed to lower the risk of BCC to date [47]. For several DNA repair gene variants such as XRCC1, XRCC3, XPA, and XPD, a significant association with BCC risk has been reported [48]. The polymorphism of those mutants involved is unfortunately reflected by diverse and often contradictory results [49–52]. A variant once proven to be significant [48] was refuted in another study [53] or was, in part, not BCC-specific at all [54]. 

Similarly unpersuasive are the results on melanocortin 1 receptor gene (MCIR), the major known genetic variant influencing the degree of skin pigmentation. Although it was clearly shown that the nonfunctional variant of MCIR had a dose-dependent impact on the incidence of BCC and melanoma, the consecutive lack in pigmentation itself did not influence the result [54, 55]. A different mechanism in terms of a paracrine role or distant modulation of proliferation and differentiation of keratinocytes by MCIR has also been suggested [56, 57]. In general, the functioning of pigmentation and DNA repair in healthy individuals, let alone in skin cancer, is so far too little, or at best partially, understood in order to pave the way for prevention or let alone treatment of BCC.

## 4. Current and Future Treatment Options

### 4.1. Current Standard of Care

A wide range of several effective therapeutic options are available for the therapy of BCC. Intended to be curative or at least locally controlling, the treatment can either be surgical or nonsurgical depending on several tumour- or patient-related factors. Especially tumour size, location, histological subtype, patient's health and wishes, possible complications, and aesthetic results should be taken into account. As there is still no preoperative method for the detection of subclinical spread, surgical therapy with 3D histology is the gold standard even in BCCs of the head and neck area. In order to ascertain the complete and hereby curative excision, several equally effective techniques are at disposal. With Mohs micrographic surgery, the histological confirmed BCC is removed in a bowl-like fashion, immediately frozen, and examined for residual tumour cells in the lateral and basal margins as long as the BCC is totally excised. 5-year recurrence rates for Mohs surgery are reported as 1%–3% for primary BCC and 3%–7% for recurrent tumours [58, 59]. Similar results are achieved with other less known histological methods such as the La Galette technique [60]. Conventional surgery with tumour-adapted margins of safety uses a bread loaf horizontal cutting to control for complete excision. Depending on the safety margin, a higher rate of residual tumour cells and thus increased recurrence rate of 4%–34% is reported [58]. Curettage, electrodesiccation, and cryosurgery are further surgical approaches that are easily applied in low-risk lesions with nonaggressive histological features such as superficial BCC of the trunk. The disadvantage is, however, that the complete removal of the BCC cannot be histological proven and delayed wound healing due to thermal destruction or impairment of the basal layer may lead to unsatisfactory results. Certainly, none of these three techniques is appropriate for recurrent or morpheaform BCCs, although in general cure rates of up to 95% and higher are stated [16]. Non-surgical treatment options include radiotherapy, photodynamic therapy, and topical application of imiquimod and 5-fluorouracil. All of the proposed procedures comprise, however, the disadvantage that no treatment success can be histologically validated and thus higher recurrence rates have to be taken into account. Nonetheless, elderly patients with multiple comorbidities and inoperable tumours profit. The indication for radiotherapy—given the multitude of therapeutic options—is more limited and rather confined to postoperative recurrences or if a complete resection appears unlikely. Since there is a high risk of secondary tumors developing on the radiation side, patients with BCNS, XP, epidermodysplasia verruciformis, and iatrogenic immunosuppression should be excluded from radiotherapy. It is also not recommended for patients younger than 60 years, given its potential for carcinogenesis [61, 62]. Photodynamic therapy requires the application of a photosensitizing agent such as 5-aminolevulinic acid or its ester 3-4 hours before the protoporphyrin IX-enriched tumour cells are destroyed. Superior with regards to cosmetic outcome when compared to many other treatment options, PDT of superficial BCC showed a 1-year recurrence rate of 9.3% [63] and is not recommended for the nodal subtype due to 5-year relapse rates of 76% [64]. Although its precise mechanism is still unknown, the once-daily application of Imiquimod 5 days per week for 6 weeks resulted in a histological clearance rate of up to 89.6% in superficial BCC [65–67]. A clear trend towards improved rates with increased frequencies of application is limited by intensified local and systemic reactions. Residual tumours after therapy are nonetheless often difficult to assess, and subtypes other than superficial BCC are no general indication for Imiquimod since multiple recurrent lesions can occur [8]. 5-fluorouracil, a topical cytostatic agent, is considered as a therapeutic alternative in patients with multiple, superficial multicentric BCCs, for example, in BCNS [68]. As a consequence of painful inflammatory and erosive reactions, the patient's compliance is often limited for this treatment option.

Given that metastasis and invasion of vital structures by BCC are extremely rare, no therapeutic “gold standard” exists. Apart from surgical procedures and an additional radiotherapy, an assortment of different chemotherapies such as doxorubicin, paclitaxel, and/or carboplatin with differing response rates have been applied up to now in order to control the tumour load and extend the patient's life expectancy [8, 69]. Given the rare nature of metastasis, larger clinical studies have been lacking until recently.

The more detailed understanding, achieved as of late, of the molecular pathogenesis of BCC and its causative aberrant pathway has rendered a new therapeutic targeted approach possible. SMO inhibitor Cyclopamine, the teratogenic steroidal plant alkaloid, which was topically applied, succeeded already to induce regression of four sporadic BCCs [70], since then, a series of small-molecule HH inhibitors have been developed and are currently in clinical development. Furthest along is GDC-0449, a more specific and potent SMO inhibitor than Cyclopamine. Administered in different doses of 150, 270, and 540 mg per day as part of a phase I study in metastatic or locally advanced BCC, an objective response in 18 of 33 patients was achieved [71]. Side effects such as hyponatraemia, fatigue, weight loss, and dyspnoea were mild to moderate. Several ongoing phase II trials investigate its efficacy in advanced BCC, medulloblastoma, and breast cancer but also in addition to other chemotherapeutic agents in pancreatic, lung, colorectal, and gastrointestinal cancer. Four other new HH inhibitors including LDE-225, BMS-833823, IPI-926, and PF-04449913 are currently under investigation in phase I trials. All of the tested components target so far SMO as the key regulator of the HH pathway. An equally effective inhibition could succeed, however, in targeting the HH downstream signalling. Two tested candidates GANT58 and GANT61, inhibitors of the GLI transcription, possibly provide a therapeutic alternative in case of a resistance to SMO inhibitors [72]. Also interfering with HH target gene transcription and therefore of potential therapeutic interest in BCC is the new field of microRNAs. While one single microRNA regulates hundreds of target genes, the task is to focus its efficacy on the essential target and thus minimize its side effects, which still needs to be mastered (Table 1) [73]. 

As discussed by Epstein, the use of tyrosine kinase inhibitors, sorafenib or imatinib, seems sensible because PDGFR*α* is supposed to mediate downstream effects in HH signalling [14]. The assortment of further new potential agents for prevention as well as treatment in BCC is plentiful, ranging from Vitamin A and D derivates, NSAID, and DNA repair enzymes up to Melanocortin peptides therapeutics. Certainly due to the lack of knowledge of their precise functioning and how, or if at all, they interact with HH pathway, results are so far promising but inconclusive. It may therefore not be surprising that even the widely recognized and in its molecular effects initially well understood standard therapy with systemic retinoids fails to prevent the recurrence of sporadic BCC [74].

## 5. Conclusion

In view of those remarkable oncogenomic achievements in the understanding of BCC carcinogenesis, a curative breakthrough, especially in hereditary, locally advanced or metastatic cases, seems imminent. First promising data are yet too scarce to obtain detailed pieces of information about dosage, side effects, response, recurrence, and survival rates or possible medical interactions. Not sufficiently known but crucial is certainly the impact of the type of HH mutations and their combinations for the various clinical subtypes of BCC. The change from a phenotype-correlated diagnosis to a genotype analysis, at least in advanced tumours, is obvious. But is BCC in all its clinical and histological variations to be reclassified according to its genotype? Genetic analysis will undoubtedly change the classification and subsequently treatment algorithms for BCC. The open intruiging question remains if there is a link between HH mutation, histology, and its clinical aspects that would simplify the indication for a targeted therapy. Of equal importance in this complex interplay are without doubt epigenetic phenomena and environmental factors that are at best only initially recognized so far. Definitely many more future studies are needed to answer the large number of interesting questions that the discovery of aberrant HH pathway for BCC raised.

## Figures and Tables

**Table 1:** Current and future HH pathway inhibitors.

SMO-Inhibitors	Ongoing trials	Indication
GDC-0449 (Erivance, Genentech)	Phase II	**BCC**, medulloblastoma, ovarian cancer, small-cell lung cancer, coloractal cancer (combined with cisplatin and etoposide), colorectal cancer (in combination with standard chemotherapy and bevacicumab), and upper gastrointestinal cancers (in combination with FOLFOLX chemotherapy)
BMS-833923 (Bristol-Myers Squibb and Exelixis)	Phase I	**BCC, BCNS**, small lung cancer (versus cisplatin and etoposide), inoperable, metastatic gastro, gastroesophageal or esophageal Adenocarcinoma (combined with cisplatin and carpecitabine), and multiple myeloma
IPI-926 Infinity Pharmaceuticals	Phase I	Advanced and/or metastatic solid tumour malignancies and metastatic pancreatic cancer (combined with gemcitabine)
LDE-225 (Novartis)	Phase I/II	Sporadic superficial and nodular skin **BCC**, **BCNS**, medulloblastoma; rhabdomyosarcoma neuroblastoma, hepatoblastoma, astrocytoma, advanced solid tumor cancers, and Medulloblastoma
PF-04449913 (Pfizer)	Phase I	select hematologic malignancies or with dasatinib in chronic myeloid leukemia (CML)
